# Corneal lacerations following crab claw injuries

**DOI:** 10.1016/j.ajoc.2022.101288

**Published:** 2022-01-20

**Authors:** Abdelhalim A. Awidi, Fasika A. Woreta

**Affiliations:** Cornea Cataract and Anterior Segment Division, The Wilmer Eye Institute, The Johns Hopkins University School of Medicine, 600 N Wolfe Street, Baltimore, MD, 21287, USA

**Keywords:** Cornea, Laceration, Crab claw, Injury, Trauma

## Abstract

**Purpose:**

To present two cases of full-thickness corneal lacerations following crab claw injuries.

**Observations:**

The first case is a 61-year-old male who presented to the emergency department (ED) with right-eye discharge, pain, and vision loss. Prior to presentation, the patient was with his friends on a fishing trip when one of them caught a crab and threw it to the patient, striking him in the right eye. The crab claw caused a penetrating injury to the cornea, with the patient presenting with a 9.5 mm full-thickness corneal laceration. He underwent emergent corneal laceration repair.

Six months post-trauma, because of the sustained injury, the patient developed a traumatic cataract. Cataract removal with an iris-sutured intraocular lens (IOL) was subsequently performed. In subsequent follow-up visits, due to the corneal scarring that developed and IOL decentration, the patient's visual acuity continued to deteriorate. This required a combined penetrating keratoplasty with IOL scleral fixation that was performed approximately three years following his initial injury date. The patient's uncorrected visual acuity (UCVA) was 20/50 and 20/80 at his one-year and two-year post-operative follow-up visits.

The second case is a 6-year-old girl who arrived at the ED with left eye conjunctival injection. Two days prior to presentation, the patient was accidently struck by a steamed crab claw while dining with her parents. She sustained a full-thickness corneal laceration injury and was diagnosed with endophthalmitis. She was taken for emergent open globe repair, anterior chamber washout, and injection of intravitreal antibiotics. Fluid taken from the anterior chamber grew *Streptococcus viridans* on culture.

A month later, following the development of a traumatic cataract and posterior synechiae, with the patient's vision decreasing to hand motions in her injured eye, she underwent cataract extraction and IOL implantation with posterior capsulectomy and PPV. Her UCVA was 20/80 and 20/60 at her two-month and four-month visits, respectively, and 20/50 at the four-year post-operative follow-up visit.

**Conclusions and importance:**

Given the settings in which both injuries occurred, awareness should be raised to handle crabs with safety precautions as they are not inherently viewed as objects that can potentially cause corneal lacerations and subsequent traumatic cataract.

## 1 Introduction

Ocular injuries are a major burden in the United States due to the potential for injuries to culminate in legal blindness (defined as visual acuity of less than 20/200) in almost one-third of serious eye injuries.^1^ Ocular injuries also pose a significant strain on the healthcare system, with $9.5 billion expended on hospital admissions and emergency department (ED) visits on them across a nine-year period.^2^

Common causes of ocular injuries that present to the ED include accidental entry of a foreign body to the eye and adnexa (29.8%), along with getting struck by or against an object (18.4%). Regardless of the cause, 37.8% of all eye injuries result in corneal abrasions and superficial laceration of the eye or its adnexal structures.^3^ Corneal lacerations may be caused by any activity where an object flies into the eye at a high speed, such as with sports-related injuries and fishing-related injuries.^4^

We present two cases in which full-thickness corneal laceration occurred following injury to the eye from a crab thrown in their direction.

## 2 Findings

### 2.1 Case 1

A 61-year-old male presented to the ED with discharge, pain, and vision loss in his right eye following trauma to the eye that occurred on a fishing trip. On that trip, a friend caught a crab and threw it to the patient; the crab claw accidently struck him in the eye. He sustained a penetrating injury to the eye and presented with a 3mm × 9.5mm full-thickness corneal laceration extending centrally to the 9 o'clock position. Loss of aqueous and uveal tissue prolapse through the wound, along with anterior capsule violation and traumatic cataract formation, was also noted. He underwent emergent repair of the corneal laceration with eleven 10-0 nylon sutures without removal of the crystalline lens.

In the consequent follow-up visits, visual acuity remained unsatisfactory to the patient, going from hand motions to light perception in his three-month and five-month post-corneal repair visits, respectively. The decision was made to perform removal of the traumatic cataract with iris suturing of a three-piece foldable acrylic intraocular lens (IOL) (MA50BM; Alcon Laboratories, Inc, Fort Worth, TX) and posterior synechiolysis at the six-month post-operative visit. Difficulties with capsulorhexis creation intra-operatively, likely due to the fibrotic lens capsule, hindered the ability to perform hydrodissection and phacoemulsification in a safe manner, leading to a pars plana vitrectomy (PPV).

**Fig. 1:**
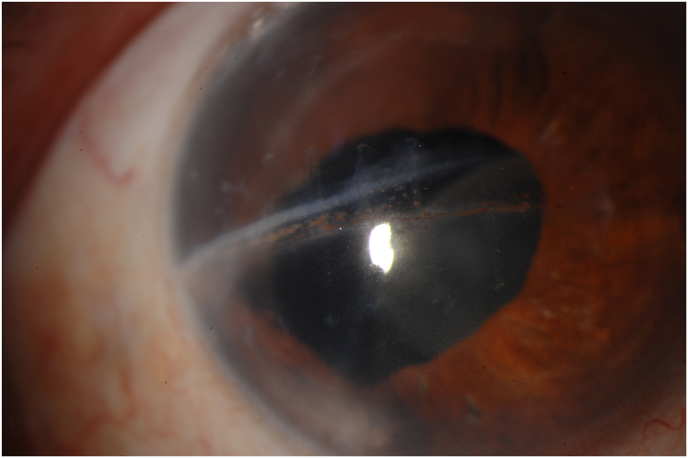
Slit lamp image showing the linear horizontal corneal scar that formed.

Three months after the combined PPV and cataract surgery, the IOL was decentered nasally. That, in combination with the linear horizontal corneal scar that formed (Fig. 1), negatively affected the patient's visual acuity. His best vision refraction was an uncorrected visual acuity (UCVA) of 20/300. Combined penetrating keratoplasty with scleral fixation of the previous IOL was performed approximately 16 months following his combined PPV and cataract surgery. The patient's UCVA improved to 20/50 by the one-year follow-up and 20/70^−1^, by his two-year post-operative follow-up visit.

### 2.2 Case 2

A 6-year-old girl presented to the ED with a left eye conjunctival injection after being struck with a steamed crab claw two days prior. The patient was dining with her parents when the crab claw was thrown in her direction, striking the left eye. On examination, the conjunctiva demonstrated 3+ injection, anterior chamber with 4+ pigmented cells, fluffy hypopyon, and a clouding view of the lens. Inspection of the cornea revealed a small full-thickness, stellate wound approximately 1–2 mm from the limbus at the 8 o'clock position. She was subsequently diagnosed with corneal laceration and endophthalmitis and was taken for emergent open-globe repair, anterior chamber washout, and injection of intravitreal antibiotics on the same day. A sample of aqueous humor had been sent for culture, which grew *Streptococcus viridans.*

A month later, the patient's vision had decreased to hand motions in her injured eye, likely due to the development of a traumatic cataract from the anterior capsule rupture and posterior synechiae. Cataract extraction with IOL implantation, posterior capsulectomy and PPV were scheduled. A three-piece foldable acrylic IOL (MA50BM; Alcon Laboratories, Inc) was implanted in the sulcus position after it was noted that 40% of the capsular bag at the 12 o'clock position was lost due to the anterior and posterior capsule fusing together. After the core vitrectomy was performed, the retina could be visualized and revealed notable lesions consistent with the history of endophthalmitis, including significant vascular changes inferiorly. The patient had no complications and recovered well, and the UCVA was 20/80 and 20/60 at her two-month and four-month post-operative visits, respectively, and 20/50 in the four-year post-operative follow-up visit.

## 3 Discussion

The risk of legal blindness in eyes with a posterior segment injury, such as retinal detachment or vitreous hemorrhage, is almost twice that of anterior segment injuries.^1^ This may explain our patients' visual rehabilitation by lack of posterior segment pathology in the first patient and prompt treatment of the posterior pathology in the second patient with PPV. In several studies, getting struck by an object, similar to our patients’ mechanism of injuries, accounted for a considerable percentage—22.7%,^5^ 31.1%,^3^ and 32.1%^2^—of all eye-related ED visits.

Open globe injuries can present with an extensive number of concomitant issues, ranging from endophthalmitis, hyphema, and retinal detachment to eyelid laceration and traumatic cataract.^2^ Traumatic cataract that develops rapidly is a common anterior segment manifestation following open-globe injuries.^2^^,^^6^ In our patients, it was a consequence of anterior capsule rupture that was brought about from direct trauma to the eye.

Both of our patients were merely bystanders when the traumatic event occurred, emphasizing the potential for crab claws to cause injuries to unsuspecting patients if not handled with caution. In a retrospective analysis of 11,320 eyes conducted by Kuhn et al.,^1^ no less than 29.6% of patients were also bystanders; the rate of blindness among all included eyes was 24.5%.

## 4 Conclusions

Given the settings that were associated with our patients’ injuries, awareness should be raised to handle crabs with safety precautions. Most people may not be familiar with how to handle crabs and the threats they may pose to bystanders. They may not associate crabs with potential corneal laceration and traumatic cataract.

## Patient consent

Consent to publish this case report has been obtained in writing from one patient and the healthcare agent of the other patient.

## Funding

No funding or grant support

## Authorship

All authors attest that they meet the current ICMJE criteria for Authorship.

## Declaration of competing interest

The following authors have no financial disclosures: (A.A., F.W.)
